# Additional psychometric data for the Spanish Modified Dental Anxiety Scale, and psychometric data for a Spanish version of the Revised Dental Beliefs Survey

**DOI:** 10.1186/1472-6831-10-12

**Published:** 2010-05-13

**Authors:** Trilby Coolidge, M Blake Hillstead, Nadia Farjo, Philip Weinstein, Susan E Coldwell

**Affiliations:** 1 Dental Public Health Sciences, University of Washington, Seattle WA, USA; 2 School of Dentistry, University of Washington, Seattle WA, USA; 3 Harvard School of Dental Medicine, Boston MA, USA

## Abstract

**Background:**

Hispanics comprise the largest ethnic minority group in the United States. Previous work with the Spanish Modified Dental Anxiety Scale (MDAS) yielded good validity, but lower test-retest reliability. We report the performance of the Spanish MDAS in a new sample, as well as the performance of the Spanish Revised Dental Beliefs Survey (R-DBS).

**Methods:**

One hundred sixty two Spanish-speaking adults attending Spanish-language church services or an Hispanic cultural festival completed questionnaires containing the Spanish MDAS, Spanish R-DBS, and dental attendance questions, and underwent a brief oral examination. Church attendees completed the questionnaire a second time, for test-retest purposes.

**Results:**

The Spanish MDAS and R-DBS were completed by 156 and 136 adults, respectively. The test-retest reliability of the Spanish MDAS was 0.83 (95% CI = 0.60-0.92). The internal reliability of the Spanish R-DBS was 0.96 (95% CI = 0.94-0.97), and the test-retest reliability was 0.86 (95% CI = 0.64-0.94). The two measures were significantly correlated (Spearman's rho = 0.38, p < 0.001). Participants who do not currently go to a dentist had significantly higher MDAS scores (t = 3.40, df = 106, p = 0.003) as well as significantly higher R-DBS scores (t = 2.21, df = 131, p = 0.029). Participants whose most recent dental visit was for pain or a problem, rather than for a check-up, scored significantly higher on both the MDAS (t = 3.00, df = 106, p = 0.003) and the R-DBS (t = 2.85, df = 92, p = 0.005). Those with high dental fear (MDAS score 19 or greater) were significantly more likely to have severe caries (Chi square = 6.644, df = 2, p = 0.036). Higher scores on the R-DBS were significantly related to having more missing teeth (Spearman's rho = 0.23, p = 0.009).

**Conclusion:**

In this sample, the test-retest reliability of the Spanish MDAS was higher. The significant relationships between dental attendance and questionnaire scores, as well as the difference in caries severity seen in those with high fear, add to the evidence of this scale's construct validity in Hispanic samples. Our results also provide evidence for the internal and test-retest reliabilities, as well as the construct validity, of the Spanish R-DBS.

## Background

Self-report measures of dental fear are commonly used to permit quick assessment of the degree of dental fear experienced by patients. Since there can be cultural differences in various anxiety disorders, including dental fear [1,2], it is important to develop measures appropriate for different cultural groups. Data from the U.S. Bureau of Census for 2008 indicate that the largest ethnic minority group in the United States is composed of Hispanics, making up approximately 15.5% of the population (statistic calculated from data presented by [3]). As part of a larger effort to reduce oral health disparities among Hispanics, researchers have pointed out that it would be useful to develop reliable and valid measures of dental fear for this population [4].

One frequently-used adult measure of dental fear is the 5-item Modified Dental Anxiety Scale (MDAS; [5]), which incorporates psychometric and content improvements over its predecessor, the 4-item Dental Anxiety Scale (DAS; [6]). The MDAS was originally developed in English, and researchers have reported good psychometric properties in various English-speaking countries such as England, Scotland, Ireland, and Wales [2,5]. Other researchers have used translated versions in additional countries, including Finland, India, China, Greece, Dubai, Brazil, and Turkey, and report that the scale appears to be reliable and valid in these countries, as well [2,7-12]. Those who score 19 or higher are considered to have high levels of dental fear [5,7,8,12].

We developed a Spanish-language version of the MDAS for use with Hispanics in the United States, and found that it had good construct validity, measured by correlations with the Spanish version of the Dental Fear Survey (DFS; [13]) in samples of Spanish-speaking students, Spanish-speaking dental patients, and Spanish-speaking adults who attended two Hispanic festivals [14]. The Spanish MDAS also displayed good criterion validity (measured by comparing questionnaire scores with observable anxiety during dental treatment) and internal reliability (measured by coefficient alpha). However, in our sample the Spanish MDAS had unexpectedly lower test-retest reliability (0.69; 95% CI = 0.34-0.85), measured by intraclass correlation in a sample of Spanish-speaking college students over a two-week interval. Thus, the first aim of this study was to re-assess the test-retest reliability of the Spanish MDAS in a new sample of Hispanic adults who were not limited to college students.

Since dental fear is often associated with avoidance behavior [15-18], a measure of dental fear should differentiate between those who go to the dentist and those who do not. Humphris et al. [2] demonstrated such a relationship with the MDAS in Ireland, Finland, and Dubai. One impact of not going to a dentist regularly is the increased likelihood of developing more serious dental problems, which may be manifested by the tendency to report that the most recent dental visit was due to pain or dental problem, compared with check-up [16,19]. Along these lines, Yuan et al. [8] found that Chinese adults who reported that their last visit was for a problem had significantly higher MDAS scores than adults whose last visit was for a check-up. However, the literature on the relationship between oral disease and dental fear is mixed, with some researchers finding that high dental fear is associated with more caries [16,17], while others do not [20]. On the other hand, greater numbers of missing teeth and fewer filled teeth are more consistently reported in fearful individuals [16,17,19,20], possibly due to a failure on the part of the fearful person to obtain dental care until the carious teeth cannot be restored and must be extracted. With these findings in mind, the second aim of our study was to further assess the construct validity of the Spanish MDAS by examining the relationships between questionnaire scores, dental attendance patterns, and oral health.

In addition to fearing specific dental procedures or stimuli, many fearful patients also express concern over the dentist's behavior [13,21]. The Dental Beliefs Survey (DBS; [22,23]) and the more recent Revised Dental Beliefs Survey (R-DBS; [13]) were designed to assess the patient's perceptions of the dentist. The R-DBS contains 28 items which are answered on a 5-point scale; possible total scores range from 28 to 140, with higher scores indicative of more negative beliefs about the dentist and treatment situation. Typical items include: "I am concerned that dentists recommend work that is not really needed", "Dental professionals say things to make me feel guilty about the way I care for my teeth", "I believe that dentists don't have enough empathy for what it is really like to be a patient", and "Once I am in the chair I feel helpless (that things are out of my control)". The R-DBS has been shown to have good reliability and validity in several samples [24-28]. Among the methods used to assess the validity of the DBS, previous authors have reported moderate correlations between the DBS and the DAS [29], and somewhat higher correlations between the DBS and DFS [30]. More recently, Coolidge et al. [26] and Abrahamsson et al. [28] have reported correlations between the R-DBS and the DAS in samples in the United States and Sweden, respectively. To date, there have been no reports on the correlation between the R-DBS and the MDAS. Furthermore, to date there have been no reports on a Spanish version of this questionnaire. Thus, the third aim of our study was to measure the correlation between the Spanish MDAS and a Spanish version of the R-DBS, as a further assessment of the construct validity of the Spanish MDAS. In addition, we report on some of the psychometric properties of the Spanish R-DBS.

In summary, in the present study we administered questionnaires and brief oral examinations to Spanish-speaking adults attending an Hispanic community festival and a church in Washington State. In this paper, we report 1) the test-retest reliability of the Spanish MDAS in a community sample; 2) the relationships between the Spanish MDAS and oral health status; and 3) the relationship between the Spanish MDAS and the Spanish R-DBS.

## Methods

The study was approved by the Institutional Review Board of the University of Washington.

### Participants

Participants were recruited at two sites during the summer of 2008. First, in order to obtain test-retest reliability, we recruited Spanish-speaking adults at a church in a suburb of Seattle, Washington. The church was chosen because it has the largest number of Spanish-speaking parishioners in the area. We also reasoned that church-goers were likely to attend the same church on subsequent Sundays, making this a potentially good location to recruit for test-retest purposes. Church staff announced the project to parishioners. Study personnel were at the church for three Sundays in one month (the fourth Sunday was a holiday and church attendance was predicted to be low on that day).

The second site was an Hispanic cultural festival held in a rural county about 100 miles from the Seattle area. The festival featured Hispanic music and dance performances, food and children's activities, as well as information booths focusing on topics related to health and education. Study personnel set up a booth on the grounds of the festival.

### Materials

We prepared a questionnaire which included the Spanish version of the MDAS used in our previous study [14]. The questionnaire also included the R-DBS, an item asking the participant if he/she currently goes to a dentist, an item asking the participant if the last visit was to treat pain/a problem or was for a check-up, demographic questions, and other items not reported here. The R-DBS and other new items were translated into Spanish and back-translated by two professional translators. Minor discrepancies were discussed by the two translators and the first author, and final decisions on wording were made by the translators. The Spanish MDAS and R-DBS items are included in Additional file 1.

### Procedures

Except for the test-retest aspect of the study, the protocol was designed so that participant data would be anonymous. At both sites, Spanish-speaking study personnel greeted potential participants and handed them a laminated card with a Spanish text describing the purpose of the study and study procedures. A few potential participants (primarily older adults at the Hispanic cultural festival) were unable to participate because they could not read the laminated card. Adults who could read the card and agreed to be in the study were given the questionnaire to fill out. Participants sat at individual desks in a classroom at the church site, and at larger tables at the festival site. Study personnel monitored the participants to make sure that they completed their questionnaires independently. After completing the questionnaire, participants were invited to participate in the dental exam. Participants received a $10 gift card for completing the questionnaire, and another $10 gift card for the dental exam. This was the end of the study procedures at the Hispanic cultural festival.

At the church site, the initial protocol was the same. However, after participants received the gift cards, they were asked if they would like to complete the questionnaire a second time on a future Sunday. They were told that we would have to ask their name if they wished to participate on another Sunday, so that we could match their two questionnaires. Those who agreed signed a consent form. On the following two Sundays of data collection, participants who were present completed the questionnaire a second time. They received another $10 gift card.

At both sites, participants who agreed to the dental examination underwent a brief oral screening using a headlight and disposable mirror only (no radiographs or probes were used). This was done in an area adjacent to the questionnaire administration and the dental personnel did not know the participants' questionnaire answers. Four examiners were trained to agreement with a gold standard (who had been previously calibrated [31]), using photographs of sound and carious teeth followed by examination of participants who were not part of this study. Third molars were excluded from consideration. The examiners rated each tooth according to WHO [32] criteria. Teeth were rated as missing if they were absent. Teeth which were present were rated as sound if there were no visible caries, having moderate caries (WHO score of D1 or D2, corresponding to visible loss of tooth substance), or having severe caries (WHO score of D3 or D4, visibly undermined enamel.) Filled teeth without visible caries were categorized as "filled", or "F". Teeth with both a filling and visible decay were rated "FD" and the level of visible caries (moderate or severe) was also rated.

### Data analyses

Questionnaire and oral health data were entered into an Excel data base using double entry to check for accuracy. If a participant selected two answers for a questionnaire item, the mean value was calculated and used in the analyses. No other changes were made to the questionnaire data. Data from the first questionnaire administration were used for all analyses except for the test-retest analyses, which used questionnaire data from both administrations. For the analyses involving the MDAS and/or R-DBS, only participants who had completed the questionnaire(s) were included. All participants who had received the oral examination were included in analyses involving missing teeth; however, only participants with at least one tooth were included in analyses involving counts of sound, carious, or filled teeth. Analyses were carried out in SPSS Version 14.0 (SPSS, Inc., Chicago, IL). Intraclass correlations (two-way, mixed) were used to examine the test-retest reliabilities of the MDAS and R-DBS. We selected SPSS' option of two-way, mixed for the test-retest analyses, which is the equivalent of Shrout and Fleiss' (3,k) model where there were k = 2 questionnaire administrations completed by each participant; this was the same option we selected in our earlier paper [33]. Cronbach's alpha was computed to examine the internal reliabilities of the two scales. Correlational analyses were used to examine the relationships between questionnaire scores, age, and numbers of carious, filled, and missing teeth. Chi square was used to assess the relationship between high dental fear (MDAS scores of 19 or higher) and presence and severity of caries. T-tests were used to compare the MDAS and R-DBS scores by gender, whether the participant goes to a dentist or not, and whether the reason for the last dental visit was to treat pain or a problem vs. a preventive visit.

## Results

Fifty nine adults participated in the study at the church site, and another 103 participated at the cultural festival. Neither age nor gender was significantly related to site, so the 162 cases were combined for the analyses. In the combined sample, the mean age was 37.75 years (SD = 12.85, range = 18-80), and the majority of participants (63%) were female. Just under half (77, or 49%) stated that they did not go to a dentist, while 80 (51%; 5 participants did not answer the question) stated that they did. One hundred fifty eight participants (97.5%) agreed to have the dental exam. Of the four who refused to be examined, one explained that he was afraid of being examined by a dentist due to a previous experience of being criticized by a dentist, while the other three did not give their reasons. One participant (.6%) was edentulous. Excluding the edentulous participant, about two-thirds (65%) of those who were examined had visible caries. The mean number of visibly carious teeth was 1.57 (SD = 2.116), and the range was 0-14. As seen in Table 1, it was more common for participants with carious teeth to have moderate caries than severe caries. Twenty eight of those examined (17.8%) had at least one tooth coded as having severe caries. About four-fifths (82.8%) had at least one filled tooth. The mean number of filled teeth was 5.61 (SD = 4.878), and the range was 0 to 18 teeth. Including the edentulous participant, about half (52.5%) of the participants who were examined had at least one missing tooth (other than third molars). The mean number of missing teeth was 2.73 (SD = 4.41); the range was 0 - 28. Most (86.1%) of those examined had at least 20 remaining teeth; the mean number of teeth present was 25.27 (SD = 4.41).

**Table 1:** Dental status of participants.

Participants with:	Number of Participants	Percent
All Teeth Sound	55	35%

Caries in One or More Teeth	102	65%

Moderate Caries	92	58.6%

Moderate Caries, but no Severe Caries	74	47.1%

Severe Caries	28	17.8%

Filled Teeth	130	82.8%

Missing Teeth	83	52.5%

Total number of participants for counts of sound, carious, and filled teeth: 157

Total number of participants for counts of missing teeth: 158

One hundred fifty six adults completed the Spanish MDAS. The mean score was 11.87 (SD = 5.10), and the range was 5 - 24. The median score was 11, with most participants scoring low (less fearful). Nineteen (12.2%) scored 19 or higher. Neither the MDAS total scores nor the classification into high vs. low fear were related to age or gender. The test-retest reliability for the MDAS was 0.83 (95% CI = 0.60-0.92), and the internal reliability was 0.88 (95% CI = 0.84-0.91).

One hundred thirty six adults completed the Spanish R-DBS. The mean score was 61.16 (SD = 25.72), with a range of 28-127. The distribution was skewed, with most participants scoring lower (fewer negative beliefs about the dentist); the median score was 61. The scores were not significantly different for males and females. However, older participants scored significantly higher on the R-DBS (Spearman's rho = 0.19, p = 0.027). On this measure, the test-retest reliability was 0.86 (95% CI = 0.64-0.94), while the internal reliability was 0.96 (95% CI = 0.94-0.97). The R-DBS was significantly correlated with the MDAS (Spearman's rho = 0.38, p < 0.001).

With regards to dental attendance, Table 2 presents the means and standard deviations for MDAS and the R-DBS for all participants, and also according to whether the participant currently goes to a dentist or not. This Table also presents the scores according to the reason for the last dental visit (preventive vs. for pain or problem). For both measures, the scores were significantly higher for those who do not go to a dentist, compared with those who do (for MDAS: t = 3.40, df = 142.501, p = 0.001; for R-DBS: t = 2.21, df = 131, p = 0.029). In addition, scores on both measures were significantly higher for those whose most recent dental visit was due to pain or another problem, compared with those whose most recent visit was for preventive care (for MDAS: t = 3.00, df = 106, p = 0.003; for R-DBS: t = 2.85, df = 92, p = 0.005).

**Table 2:** Means, standard deviations, and numbers of participants on MDAS and R-DBS for all participants, and significances of mean differences on MDAS and R-DBS by dental attendance.

	MDAS	MDAS	R-DBS	R-DBS
	Mean (N)	SD	Mean (N)	SD
Overall Scores	11.87 (156)	5.10	61.16 (136)	25.72

Currently Goes to Dentist?				
Yes	10.54 (78)	4.41	55.75 (69)	24.92
	
No	13.26^a ^(75)	5.43	65.27^b ^(64)	24.73

Reason for Last Visit?				
Preventive	10.39 (74)	4.30	54.11 (64)	22.09
	
Pain/Problem	13.19^c ^(34)	4.94	68.88^d ^(30)	26.15

^a ^MDAS comparison between those who do and do not go to a dentist: p = 0.001

^b ^R-DBS comparison between those who do and do not go to a dentist: p = 0.029

^c ^MDAS comparison between those for whom last dental visit was for preventive care vs. pain/problem: p = 0.003

^d ^R-DBS comparison between those for whom last dental visit was for preventive care vs. pain/problem: p = 0.005

Table 3 displays the number and percentage of participants with high (MDAS 19 or greater) and low fear (MDAS 18 or lower), according to whether they had all sound teeth, only moderate caries, or severe caries. The distribution of high vs. low fear by caries states was significant (Chi-square = 6.64, df = 2, p = 0.036). However, there were no significant correlations between MDAS total scores and the number of carious, filled, or missing teeth.

**Table 3:** Number and percent of participants by caries status and by high and low dental fear.


	**Number of Participants with All Teeth Sound (Percent)**	**Number of Participants with Only Moderate Caries Present (Percent)**	**Number of Participants with Severe Caries Present (Percent)**

Participants with High Fear (MDAS >= 19)	6 (10.9%)	6 (8.5%)	7 (28%)

Participants with Low Fear (MDAS < 19)	49 (89.1%)	65 (91.5%)	18^a ^(72%)

^a ^MDAS comparison between caries status and high vs. low dental fear: p = 0.036

Turning to the R-DBS, there were no significant relationships between total scores on this measure and the number of carious or filled teeth. On the other hand, higher R-DBS scores were significantly related to having a greater number of missing teeth (Spearman's rho = 0.23, p = 0.009). This relationship remained significant after controlling for age (Pearson's r = 0.22, p = 0.012).

## Discussion

The primary aim of this study was to reassess the test-retest reliability of the Spanish MDAS. We had obtained a test-retest statistic of 0.69 (95% CI = 0.34-0.85) for this measure in our previous study, which used a sample of Spanish-speaking college students to assess this [14]. As reported in our earlier paper, the relatively low test-retest statistic we obtained puzzled us as we had obtained a higher value for the MDAS with an English-speaking student sample (0.94; 95% CI = 0.87-0.97). Furthermore, two other Spanish measures we had tested in the Spanish student sample had yielded good test-retest results (0.86 and 0.94; 95% CIs = 0.68-0.97 and 0.86-0.97, respectively); these values were also similar to the results obtained for the same two measures in the English-speaking student sample (0.94 [95% CI = 0.87-0.97] and 0.88 [95% CI = 0.73-0.95], respectively). The test-retest reliability of the Spanish MDAS was higher in this sample. (0.83; 95% CI = 0.60-0.92). This value is considered "excellent", according to Fleiss' criteria [34]. While neither the previous study nor the current study used representative samples of Hispanics, the community participants surveyed in the current study are likely to be more typical of Hispanic adults than the previous sample of college students, and thus our obtained test-retest value may be more similar to what would be expected of a representative sample of Hispanics.

Consistent with previous reports [2,8,15-19], participants in our sample with higher dental fear are less likely to currently go to a dentist, and are more likely to report that their last dental visit was to treat pain or another dental problem, rather than a routine check-up. These findings provide additional data for the construct validity of the Spanish MDAS. We also found that the Spanish MDAS and the Spanish R-DBS were moderately correlated. Given that others have found significant correlations between the DAS and either the DBS or the R-DBS [26,28,29], we believe that our results represent additional evidence for the construct validity of the Spanish MDAS.

The moderate correlation between the Spanish MDAS and R-DBS also indicates that the two scales are tapping somewhat similar, but not identical, underlying constructs. For example, it might be possible for individuals to be highly fearful of certain dental procedures, assessed by the MDAS, but not necessarily to have negative beliefs about the dentist, assessed by the R-DBS. For this reason, we believe that our results could also be used to document psychometric properties of the Spanish R-DBS. Specifically, our results indicate that the Spanish R-DBS appears to have good internal (0.96) and test-retest (0.86) reliabilities. In addition, the findings that participants with more negative beliefs about dentists are less likely to be seeing a dentist currently, and more likely to have had treatment for pain or a problem (rather than a check-up) at their last visit, provide evidence for the construct validity of the Spanish version of this questionnaire.

The relationships between questionnaire scores and oral health status were different for the two measures. While we did not find a significant correlation between fear and the number of carious teeth, we did find that being classified as having high dental fear was associated with having severe caries. However, the R-DBS was not related to caries. On the other hand, we found that there was a significant correlation between negative beliefs about the dentist and the number of missing teeth, while there was no relationship between dental fear and missing teeth. As noted previously, the reports of other authors have been inconsistent in the relationships between dental fear and caries [16,20]. It is possible that the differing results are related to culture, age, and/or factors related to the representativeness of the various samples. Further, the differing results we found are likely to be related to the fact that the constructs underlying the two scales are somewhat different.

We found no relationship between MDAS or R-DBS scores and gender in this study, which differs from other studies which often find that women have significantly greater dental fear than men. In our previous study, we did not find gender differences on either the MDAS or the DFS in either the student or community festival samples [data computed but not reported in [14]]. We also did not find gender differences on these measures in the sample of 100 Spanish-speaking patients at a dental office [35]. Turning to literature using Spanish versions of the original DAS and/or versions of the DFS, the initial report of a Spanish version of the DAS for Costa Rica stated that there were no significant gender differences in a convenience sample of 158 adolescents aged 14-17 [36]. A subsequent study, using the same version of the DAS as in the initial report but in a representative sample of adults living in one area in Costa Rica, reported that women scored significantly higher than men [37]. A third study reported no gender differences with either this version of the DAS, or a Spanish version of the DFS, in a sample of 70 adults in Spain who completed the questionnaires at a consultation appointment before hearing information about a recommended third molar extraction [38]. A fourth study reported that men and women had equal levels of fear on a Spanish version of an older form of the DFS, in a study of 253 patients in Spain [39]. Finally, a fifth study reported no gender differences on a Spanish version of the DAS (no information provided about the source of the version) in a sample of 76 dental patients in Spain [40]. It is hard to know whether our failure to find significant gender differences is related to the fact that we did not utilize a representative sample (as in the one study that found significant gender differences on a Spanish version of the DAS), or whether for unknown cultural reasons Spanish-speaking men often score similarly to Spanish-speaking women on measures of dental fear. Since the gender results in the Spanish samples are, by and large, different from what has been reported in other studies, this bears further exploration.

In this study, we used samples of convenience to assess the relationships between measures of dental fear (MDAS), negative perceptions of the dentist (R-DBS), dental attendance, and oral health status in Spanish-speaking adults. Since most Hispanics in Washington State are Mexican-American, in order to consider the possible generalizability of our findings it is instructive to compare the oral health status of our participants with data derived from representative regional and national samples of Mexican-Americans. For example, we found that 0.6% of our participants were edentulous, while 86.1% had 20 or more teeth (excluding third molars). Using data collected from 1988 to 1994 in the National Health and Nutrition Examination Survey (NHANES III), Jimenez et al. [41] reported that 5% of the Mexican-American adults were edentulous and 83% had 20 or more teeth (excluding third molars). The participants in our study had means of 1.57 carious teeth, 5.61 filled teeth, and 25.27 remaining teeth, excluding third molars. Researchers analyzing data from southwestern Mexican-American adults in the Hispanic Health and Nutrition Examination Survey of 1982-1984 (HHANES) reported means of 1.46 carious teeth (statistic calculated from data presented in [42]), 4.35 filled teeth (statistic calculated from data presented in [42]), and 27.2 remaining teeth ([43]; statistics include third molars if present). These similarities suggest that our study findings are likely to generalize to other Hispanics who are Mexican-Americans. However, it should be noted that oral health data for Hispanics show changes over time, likely due to increased assimilation. For example, Wall and Brown [44] compared National Health Interview Survey data from 1978-1980 and 1999, which revealed that the percent of Mexican-Americans over age 4 who had visited a dentist in the previous 12 months increased from 44.7 to 59.3. Thus, it would be useful to compare our results with more contemporary representative samples. In addition, further studies would need to be carried out in order to establish the norms of the Spanish MDAS and R-DBS.

Jimenez et al.'s [41] report includes data that bear relevance to the relationship between cultural group, dental fear, and dental status. They report large differences between U.S. Hispanics and other U.S. residents in terms of their dentate status: while 70% of U.S. residents in general, 63% of Caucasians, and 68% of African Americans have 20 or more teeth, 83% of Hispanic residents of the U.S. have 20 or more teeth. Since our previous work [14] found that Spanish- and English-speaking attendees at two Hispanic community festivals had similar scores on the MDAS (i.e., means of 13.05 for the Spanish version and 12.64 for the original English version), the relationship between dental fear and tooth loss may be different for Hispanics than what has been previously reported for other populations.

It is important to note that our protocol required a written questionnaire, which means that potential participants of low literacy could not be included. Similar to what we described in our previous paper [14], we encountered a few interested adults who could not participate in our study due to low literacy. More inclusive estimates of dental fear in Hispanics might require alternative (non-written) methods appropriate for this population [45]. Furthermore, our cross-sectional design limits us to reporting the associations between variables, and does not provide evidence for causal relationships between them.

## Conclusion

In this study, we found good evidence for the test-retest reliability for the Spanish MDAS in community samples, as well as additional evidence for its construct validity. We also found good evidence for the reliability and construct validity of the Spanish R-DBS in community samples.

## Competing interests

The authors declare that they have no competing interests.

## Authors' contributions

TC designed the study, with the assistance of PW and SEC. MBH and NF collected and entered data, and performed the preliminary data analyses. TC oversaw the translation and data collection activities, performed the final data analyses, and wrote the manuscript. All authors read and approved the manuscript.

## Pre-publication history

The pre-publication history for this paper can be accessed here:

http://www.biomedcentral.com/1472-6831/10/12/prepub

## Supplementary Material

Additional file 1 **Spanish Language Questionnaires**. This file contains the Spanish versions of the Modified Dental Anxiety Scale (Spanish MDAS) and the Revised Dental Beliefs Survey (Spanish R-DBS) Click here for file
