# Supplementary material for: Additional psychometric data for the Spanish Modified Dental Anxiety Scale, and psychometric data for a Spanish version of the Revised Dental Beliefs Survey
Source: BMC Oral Health. 2010 May 13;10:12. doi: 10.1186/1472-6831-10-12 (PMC2887771; doi:10.1186/1472-6831-10-12)
Supplement: Additional file 1 — Spanish Language Questionnaires. This file contains the Spanish versions of the Modified Dental Anxiety Scale (Spanish MDAS) and the Revised Dental Beliefs Survey (Spanish R-DBS) [file 1472-6831-10-12-S1.DOC]

Additional File 1

Spanish Modified Dental Anxiety Scale (Spanish MDAS)

Por favor indique lo ansioso que se pone, si es que se pone, con su visita dental. Por favor, ponga un círculo alrededor de un número para cada pregunta.

1 = Sin ansiedad

2 = Levemente

3 = Bastante ansioso

4 = Muy ansioso

5 = Extremadamente ansioso

1. ¿Cόmo se sentiría si tuviera que ir a su dentista para un tratamiento mañana?

2. ¿Cómo se sentiría si estuviera sentado/a en la sala de espera (esperando por el tratamiento)?

3. ¿Cómo se sentiría si estuvieran a punto de agujerarle un diente?

4. ¿Cómo se sentiría si estuvieran a punto de quitarle el sarro de los dientes y pulírselos?

5. ¿Cómo se sentiría si estuvieran a punto de ponerle una inyección de anestesia local en su encía, sobre uno de los dientes de arriba de la parte de atrás de su boca?

Spanish Revised Dental Beliefs Survey (Spanish R-DBS)

Los siguientes numerales, en este cuestionario, se refieren a varias situaciones, sentimeintos, y reacciones relacionadas al trabajo dental. Por favor califica tus sentimientos o creencias respecto a estos enunciados colocando un círculo alrededor del número (1, 2, 3, 4 o 5) de la categoría que más se aproxima a los sentimientos que tienes por la dentistería en general.

1 = Nunca

2 = Una o dos veces

3 = Unas pocas veces

4 = Frecuentemente

5 = Casi siempre

1. Me preocupa que los dentistas recomienden tratamientos que realmente no se necesitan.

2. Yo creo que los dentistas hacen o dicen cosas paraocultarme información.

3. Me preocupo si el dentista es técnicamente competente y si está hacienda trabajo de calidad.

4. He tenido dentistas que dicen una cosa y hacen otra.

5. Me preocupa que el dentista me proporcione toda la información que necesito para tomar buenas decisiones.

6. A los dentistas parece no importarles que los pacientes necesiten descansar a veces.

7. He tenido dentistas parecer renuentes a corregir trabajos que no han sido satisfactorios para mi.

8. Cuando un dentista parece estar de afán me preocupa no estar recibiendo un cuidado adecuado.

9. Me preocupa que el dentista realmente no esté buscando lo mejor de acuerdo a mis intereses.

10. Los dentistas se concentran mucho en terminar el trabajo y no lo suficiente en la comodidad del paciente.

11. Me preocupa que los dentistas no tengan la habilidad suficiente para manejar mis temores o problemas dentales.

12. Siento que los dentistas no dan explicaciones claras.

13. Me preocupa que a los dentistas no les gusta tomarse el tiempo para realmente hablarle al paciente.

14. Me siento incómodo hacienda preguntas.

15. Los profesionales dentales dicen cosas para hacerme sentir culpable por la forma como cuido mis dientes.

16. Me preocupa que los dentistas no tomen seriamente mis preocupaciones (temores) hacia la dentistería.

17. Me preocupa que los dentistas me hagen sentir mal (no tomen en serio mis temores).

18. Me preocupa que los dentistas no les guste cuando un paciente hace una petición.

19. Me preocupa que el personal dental me avergüence por la condición de mis dientes.

20. Yo creo que los dentistas no tienen suficiente empatía por lo que implica realmente ser un paciente.

21. Cuando estoy en el sillón dental me siento incapaz de detener las consulta para descansar, si siento la necesidad.

22. Los dentistas parecen no notar que los pacientes necesitan descansar a veces.

23. Una vez estoy en el sillón dental me siento indefenso (porque las cosas están fuera de mi control).

24. Si yo fuera a indicar que me duele, pienso que el dentista va a ser renuente para detenerse y tratar de corregir el problema.

25. He tenido dentistas que no me creen cuando he dicho que he sentido dolor.

26. Los dentistas frecuentemente parecen estar apurados, entonces yo me siento afanado.

27. Me preocupa que el dentista haga lo que él quiera y realmente no me escuche cuando estoy en el sillón.

28. Sentirme abrumado por la cantidad de trabajo que necesito (todas las malas noticias) es suficiente para evitar tratamiento.
